# Management of Craniocervical Instability in Spondyloepiphyseal Dysplasia Congenita: Assessment of Literature and Presentation of Two Cases

**DOI:** 10.7759/cureus.27020

**Published:** 2022-07-19

**Authors:** Cody J Falls, Paul S Page, Garret P Greeneway, James A Stadler

**Affiliations:** 1 Orthopedic Surgery, Baylor University Medical Center, Dallas, USA; 2 Neurological Surgery, University of Wisconsin, Madison, USA; 3 Neurosurgery, University of Wisconsin Hospitals and Clinics, Madison, USA; 4 Neurological Surgery, University of Wisconsin School of Medicine and Public Health, Madison, USA

**Keywords:** sedc, pediatric spinal anomalies, pediatric spine, atlantoaxial instability, spine

## Abstract

Spondyloepiphyseal dysplasia congenita (SEDC) is a rare autosomal dominant skeletal dysplasia resulting in impairment of type II collagen function. Phenotypically, this results in various skeletal, ligamentous, ocular, and otologic abnormalities. Platyspondyly, scoliosis, ligamental laxity, and odontoid hypoplasia are common, resulting in myelopathy in a high number of patients due to atlantoaxial instability. Despite patients undergoing surgical fixation, complication rates such as nonunion have been reported to be high. Here within, we present two patients treated with occipitocervical fusion for atlantoaxial instability and early symptoms of progressive myelopathy. We additionally provide a detailed review of the literature to inform practitioners of the spinal manifestations and clinical considerations in SEDC.

## Introduction

Spondyloepiphyseal dysplasia congenita (SEDC), originally described by Spranger and Wiedemann in 1966, is a rare skeletal dysplasia resulting from a defect in the COL2A1 gene, the gene encoding for the alpha-1 chain of type II collagen [1,2]. Various mutations, which may be de novo or inherited in an autosomal dominant fashion, have been described - all of which result in impairment of type II collagen function and thus skeletal, ligamentous, ocular, and otologic abnormalities [3]. Characteristic features include dwarfism, flattened facies, waddling gait (secondary to bilateral coxa vara), scoliosis, and ligamentous laxity. At the craniocervical junction, SEDC patients commonly exhibit odontoid hypoplasia and/or os odontoideum resulting in atlantoaxial instability. With myelopathy rates as high as 35% in these patients, craniocervical stabilization via operative fixation is frequently employed given concern for progressive instability and risk for myelopathy [1,4-6].

Here within, we provide two descriptive cases of patients who presented with significant atlantoaxial instability. Both patients underwent unilateral occipitocervical fusion and have maintained craniocervical stability without onset of new pathology stemming from the craniocervical junction (CCJ). We additionally provide an extensive review of the current literature regarding the operative management of CCJ pathology in SEDC with an aim to guide practitioners who are tasked with the management of these patients.

## Case presentation

Case 1

Presentation

**Figure 1:**
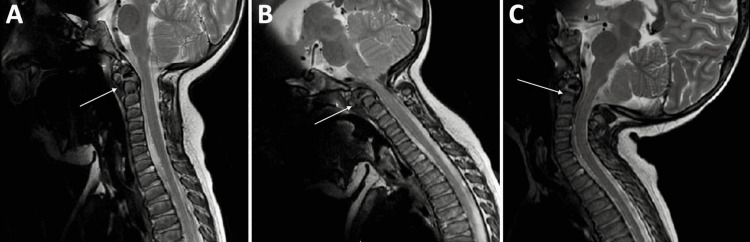
Preoperative T2 weighted MRI sagittal sequences demonstrating dynamic instability and compression of the upper cervical spinal cord on flexion and extension positioning (arrow showing site of maximal compression). A: Neutral Position, B: Flexion, C: Extension.

A five-year-old girl was referred to us by her pediatrician due to abnormal spinal imaging in the setting of SEDC (genetically confirmed). She endorsed no associated symptoms, and her physical exam was unremarkable. A review of spinal imaging revealed flexion-extension c-spine plain films demonstrating significant atlantoaxial instability. At this time, activity restrictions were implemented and flexion-extension MRI studies were ordered. Flexion-extension MRI revealed atlantoaxial instability with the widening of the atlanto-dens interval from 5mm on flexion to 2mm in extension (Figure 1). Anterior to posterior (AP) spinal canal diameter at the C1-C2 articulation was also narrow measuring 9mm in flexion, 11mm neutral, and 12mm in extension. Hypoplasia of the C1 vertebrae presents with incomplete formation of the posterior arch. Of particular importance, T2 signal changes were apparent within the cord indicating early myelopathy. With these findings, occipitocervical (OC) fusion was recommended.

Surgical Intervention

**Figure 2:**
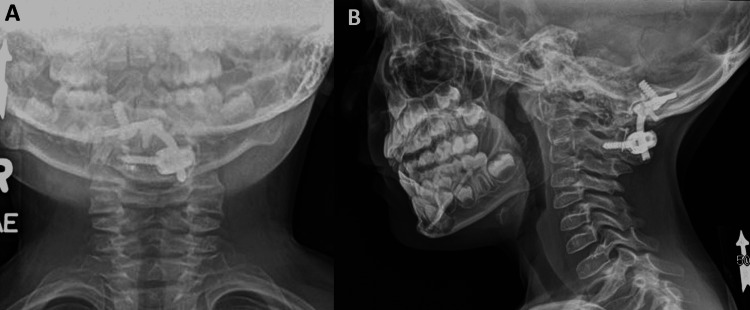
AP and lateral radiographs demonstrating occipital to C2 instrumentation with a unilateral occipital plate and unilateral C2 translaminar screw placement. AP: Anterior to posterior

At five years of age, the patient underwent OC fusion via rigid posterior instrumentation. Occipitocervical fusion was decided upon given the abnormality anatomy at C1. The operation entailed right-sided C2 translaminar screw placement which was connected to an occipital plate with a unitized rod. Given the hypoplastic nature of C1, no instrumentation was able to be utilized at that level. After careful arthrodesis of the posterior C1, C2, and occiput elements, rib graft harvested earlier in the procedure was utilized as a strut between the C2 posterior arch and the occiput to maximize C1 incorporation into the construct. This strut was secured with titanium cranial plates and screws (Figure 2). Non-structural allograft and bone morphogenic protein were used to augment the fusion construct.

Postoperative Care

Postoperatively, the patient was placed in a custom-fitted pinless halo brace to be worn at all times for six months. There were no complications during her hospital stay and she was discharged on postop day 2. At three months postop, AP and lateral plain films demonstrated appropriate bony incorporation and stable instrumentation. At 2.5 years postop she continues to do well with an excellent cervical range of motion (85 degrees rotation bilaterally, full flexion/extension), normal neurological exam findings, and no new complaints.

Case 2

Presentation

**Figure 3:**
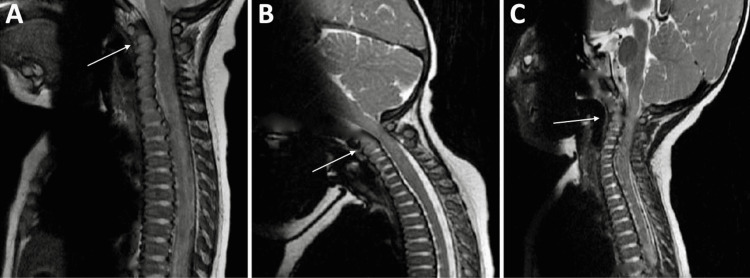
Preoperative T2 weighted MRI sagittal sequences demonstrating dynamic instability and compression of the upper cervical spinal cord on flexion and extension positioning (arrow demonstrating site of maximal compression). A: Neutral Position, B: Flexion, C: Extension.

A nine-month-old male was referred for the concern of craniocervical instability in the setting of SEDC (genetically confirmed) following an abnormal sleep study. The patient’s parents endorsed no apparent symptoms and reported he was within the normal range for all developmental milestones aside from being able to sit up unassisted. The physical exam was normal without signs of myelopathy. Sleep studies were reviewed, which showed mixed sleep apnea with a more prominent central component. At this point, flexion-extension MRI was recommended. Flexion-extension MRI revealed a hypoplastic C1 vertebra and significant craniocervical joint laxity leading to substantial spinal canal narrowing at C1/C2 (6.5mm in flexion, 10mm in neutral and extension). Flexion views demonstrated circumferential loss of cerebrospinal fluid signal in this area. There was no appreciable change in spinal cord T2 signal change (Figure 3).

Due to the patient’s immature osteology and lack of symptoms, conservative management with a cervical collar to be worn during activity was recommended. It was discussed that the patient may need decompression and craniocervical stabilization via OC fusion in the future, ideally at an interval that has allowed sufficient time for maximal bony maturation. The patient was followed over the following three years where he continued to develop normally, showed improvement in his sleep studies, and showed no new myelopathic symptoms. Imaging three years from his original visit showed progressive cervical lordosis and continued craniocervical instability. With these findings, surgery was recommended.

Surgical Intervention

At four years of age, the patient underwent OC fusion. This procedure entailed rigid posterior instrumentation with the placement of a unilateral translaminar screw at C2 secured to the occipital plate via a unitized rod and reduction/nonrigid instrumentation of C1 via a thick braided suture ran in the sublaminar area to the rod. Careful arthrodesis of the posterior elements of occiput, C1, and C2 was then performed and the fusion surface was augmented with nonstructural allograft and bone morphogenic protein. Direct decompression was not necessary as it reduced appropriately.

Postoperative Care

Postoperatively, the patient was custom-fitted pinless halo brace, with plans to be worn at all times for six months. There were no complications during the patient’s postoperative period and he was discharged on postop day 6. At six months postop, the patient continues to do well with no symptoms or signs of myelopathy. Cervical plain films at this visit demonstrated good CCJ stability with apparent bony incorporation.

## Discussion

Background

SEDC is a type of skeletal dysplasia first described by Spranger and Wiedemann in a series of six patients who presented with a heritable bone dysplasia with disproportionately short trunks [1]. The name SEDC was appropriately chosen as the most substantial abnormalities were of the spine and proximal epiphyses with these abnormalities present at birth. The disorder is quite rare with an incidence of 1:100,000 per year [7]. There are numerous characteristic features that present with SEDC as it is a result of defective type II collagen production, a ubiquitous fibrillar protein essential to the normal functioning/development of various organ systems. Common findings include myopia, retinal hemorrhage/detachment, hearing loss, cleft palate, scoliosis, kyphosis/lordosis, coxa vara, genu varum/valgum, craniocervical instability, characteristic facial features (flat facial profile, upward slanting of palpebral fissures, frontal bossing) craniovertebral stenosis, and various joint abnormalities [1,8].

Craniocervical instability

Though the ocular and otologic manifestations of the disorder are undoubtedly a great source of morbidity, the associated skeletal manifestations - particularly of the cranium and spine - are perhaps the most concerning as they can lead to acute neurologic deterioration if improperly managed. These patients exhibit high rates of craniocervical instability due to a combination of their highly lax ligaments as well as the high prevalence of odontoid hypoplasia and/or atlantoaxial instability secondary to os odontoideum. This is particularly worrisome as these patients show a high propensity to subluxate at the atlantoaxial joint with subsequent cervical myelopathy. Previous studies have demonstrated myelopathy rates as high as 35% in these patients, with those displaying extremely short stature (-7 SDs) and severe coxa vara being at particularly high risk [1,4,5,9]. Myelopathic symptoms often present subtly and/or may be obscured by the clinical picture of SEDC which has pushed some authors to propose for annual cervical screening [10].

Genetics

SEDC is one of the various “type 2 collagenopathies”, a group of disorders characterized by defective type 2 collagen production. COL2A1 is the gene affected in this disorder, most commonly being caused by a heterozygous missense mutation resulting in a glycine to serine substitution in the triple-helical region [3]. Though this is the most common mutation, various other mutations within the 54 exons of the COL2A1 gene may result in SEDC, with 539 mutations being identified to date [11]. The disorder is most commonly de novo though it may be inherited in an autosomal dominant fashion [1,3]. As there is vast phenotypic overlap in the type 2 collagenopathies and the prognosis/management of these disorders may greatly differ, molecular genetic confirmation testing should be carried out in all suspected individuals [12-14].

Assessment of literature

An assessment of literature was carried out revealing a total of 53 cases from nine authors (Table 1). Of the 53 cases, 18 presented with myelopathy (34%) with postsurgical improvement of symptoms in 15 of 18 cases (83%). Odontoid hypoplasia was present in 19 cases (36%) and os odontoideum was present in 11 (21%). The mean patient age at the time of surgery (excluding cases where individual ages were not provided) was 13 years and the median age was 8 with an age range of two years to 56 years. Eighty-nine percent of the operations were performed in pediatric patients with 67% of those being eight years old or younger. Twenty of the cases were male patients, whereas 32 were female patients, one patient's sex was not provided. Various surgical techniques were employed with some cases requiring modifications due to anatomic abnormalities/size (Table 2). Notably, of the cases that provided surgical details, only three cases employed rigid-only instrumentation. Most authors utilized non-rigid instrumentation (16 cases) with two cases utilizing rigid posterior instrumentation with non-rigid instrumentation to capture a hypoplastic C1 within the construct. Occipital to C2 instrumentation was by far the most common method of craniocervical stabilization (72%) followed by O-C3 (12%), and C1-C2 (9%). Bony fusion was achieved in 37 cases, was not achieved in four cases, and was not reported in 12 cases. Of the four cases that did not achieve bony fusion, three were in non-instrumented in situ fusion patients, and one was with a non-rigid construct. Two of these four cases achieved stable non-union and two required revision surgeries. The most common procedure that occurred along with posterior fusion was C1 laminectomy/decompression for narrowing of the spinal canal, occurring in 20 cases. No patients have developed myelopathy after craniocervical stabilization with a mean follow-up period of 71.9 months (range: 3-225 months).

**Table 1:** Review of operative cases addressing craniocervical instability in SEDC patients with case details. -- = Details not available, C = cervical vertebra, F = female, M = male, NA = Not Applicable, NR = Not reported, O = Occipital, OO = Os odontoideum, OH = Odontoid hypoplasia, Pt = Patient, mo = month, yr = year, Sx = symptom *Authors' stated stability was achieved through fibrous union, **Radiographic evidence of early myelopathy.

Authors, Year published	Patient Age/Sex (Age at operation)	Genetically confirmed?	Odontoid hypoplasia (OH) or Os odontoideum (OO)?	Myelopathic Sx (+/-)	Rigid (R)/non-rigid (NR) instrumentation, levels instrumented, other procedures done	Bony fusion? (Y/N)	Improvement of Sx?, New-onset Sx?	Follow-up
Svensson and Aaro, 1988 [14]	1. M, 3; 2. M, 6; 3. --, 8	No (all cases)	1. --, 2. --, 3. OH	1. +, 2. -, 3. -	1. NR, O-C2; 2. --, O-C2; 3. --, O-C2	Y (all cases)	1. Yes, no; 2. NA, no; 3. NA, no	1. 2yrs, 2. 7yrs, 3. 2.5yrs
LeDoux et al., 1991 [15]	M, 4	No	--	-	R/NR, C1-C4 on right/C2-C5 on left, Sublaminar wiring of C1 to C2 (Interlaminar clamps used)	--	NA, no	--
Gembun et al., 2001 [16]	M, 56	No	OO	+	R, O-C2, C1 laminectomy	Y	Yes, no	8mos
Miyoshi et al., 2004 [4]	1. F, 8; 2. F, 8; 3. F, 8; 4. F, 25; 5. M, 33; 6. M, 35; 7. M, 48	No (all cases)	OO (all cases)	1. +, 2. +, 3. +, 4. +, 5. +, 6. +, 7. +	1. NR, O-C2, C2 laminoplasty; 2. NR, O-C2, C2 laminoplasty/ enlargement of foramen magnum; 3. NR, O-C2, C2 laminoplasty/ enlargement of foramen magnum, duraplasty; 4. NR, O-C2, Enlargement of foramen magnum; 5. NR, O-C2, Enlargement of foramen magnum; 6. NR, O-C2, C2 laminoplasty/enlargement of foramen magnum, duraplasty; 7. NR, O-C2, C2 laminoplasty. (All pts underwent C1 laminectomy)	1. N*, 2. Y, 3. Y, 4. Y, 5. Y, 6. Y, 7. Y	1. Yes, --; 2. Yes, --; 3. No, --; 4. Yes, --; 5. Yes, --; 6. Yes, --; 7. No, --	Avg. follow-up of 42.4 mos (range, 18-92mos)
Veeravagu et al., 2013 [17]	M, 45	--	OH	+	R, C1-C3, suboccipital craniotomy/decompression of foramen magnum, arch of C1, lamina of C2.	--	Yes, no	1yr
Sitoula et al., 2014 [18]	1. F, 2; 2. F, 5; 3. F, 2; 4. F, 4; 5. M, 3; 6. M, 8; 7. F, 3; 8. F, 3	--	1. OH, 2. OH, 3. OH, 4. OH, 5. OO, 6. OH, 7. OH, 8. OO	1. -, 2. -, 3. -, 4. -, 5. -, 6. -, 7. -, 8. -	1. NR, O-C3; 2. NR, O-C2; 3. NR, O-C2; 4. NR, O-C2; 5. NR, O-C2; 6. NR, O-C2; 7. NR, O-C2; 8. NR, O-C3 (All cases except pt 1. underwent C1 decompression, Pt. 6 also underwent foramen magnum decompression)	Y (for all)	NA, no (all cases)	1. 6.8yrs, 2. 2.8yrs, 3. 2.9yrs, 4. 2.6yrs, 5. 2.7yrs, 6. 8.2yrs, 7. 6.1yrs, 8. 5.5yrs
Serhan Er et al., 2017 [19]	20 children (17 F, 3 M)	--	--	+ (3/20), - (17/ 20)	Both R & NR (individual case details not provided); 15 cases instrumented, 5 cases in situ fusion; O-C2 (12), O-C3 (3), C1-C2 (4), O-C5 (1)	Y (17/20), N (3/20) (All cases of bony non-fusion occurred in in situ fusions)	NA, no (17/20); Yes, no (2/20); No, no (1/20)	Mean: 104mos. Range: 33-225mo
Al Kaissi et al., 2019 [10]	1. M, 17; 2. M, 5; 3. M, 3; 4. F, 8; 5. F, 6; 6. M, 10; 7. M, 16; 8. M, 13; 9. F, 10; 10. F, 7	Yes (all cases)	1. OO, 2. OH, 3. OH, 4. OH, 5. OH, 6. OH, 7. OH, 8. OH, 9. OH, 10. OH	1. +, 2. +, 3. +, 4. -, 5. +, 6. -, 7. -, 8. -, 9. -, 10. -	-- (Pts 1, 2 & 3 underwent C1 laminectomy)	--	1. Yes, no; 2. Yes, no; 3. Yes, no; 4. NA, --; 5. --,--; 6. NA, --; 7. NA, --; 8. NA, --; 9. NA, --; 10. NA, --	--
Falls et al., (Current report)	1. F, 5; 2. M, 4	Yes (both cases)	1. OH, 2. OH	1. + **, 2. -	1. R- O-C2; 2. R/NR- O-C2, sublaminar wiring of C1 to rod	1. Y, 2. Y	1. NA, no; 2. NA, no	1. 2.5yrs, 2. 6mos

**Table 2:** Anatomic abnormalities faced during surgery with methods of management and complications. SCD = Spinal canal diameter, FM = Foramen magnum, --- = Individual case details not provided

Authors, Year published	Anatomic abnormality encountered	Method of management	Complications
LeDoux et al., 1991 [15]	C1 posterior arch midline defect	Sublaminar wiring of C1 to C2 for inclusion in fusion mass	None
Gembun et al., 2001 [16]	Narrow C1 SCD	C1 laminectomy	None
Miyoshi et al., 2004 [4]	1. Narrow C1 SCD, 2. Obstruction of reduction position, 3. Constricting dural band at C1 laminectomy site.	1. C1 laminectomy, 2. Enlargement of FM/ C2 laminoplasty, 3. Duraplasty at C1	1. None, 2. None, 3. None
Veeravagu et al., 2013 [17]	SCD narrowing at C1/C2, narrow FM	Posterior decompression of FM, C1, C2	None
Sitoula et al., 2014 [18]	SCD narrowing at C1, narrow FM	Posterior decompression of C1, FM	None
Serhan Er et al., 2017 [19]	SCD narrowing at C1	C1 decompression	---
Al Kaissi et al., 2019 [10]	SCD narrowing at C1	C1 laminectomy	None
Falls et al. (Current report)	1. Small C2 vertebrae, 2. Hypoplastic C1 posterior arch	1. Unilateral instrumentation, 2. Sublaminar wiring of C1 to posterior rod for inclusion in fusion mass.	1. None, 2. None

This data further supports the utility of operative craniocervical stabilization in SEDC patients to prevent myelopathy. No clearly superior method was discerned from the assessment of this data so long as stabilization was achieved. Myelopathy was noted in patients as young as three years of age, demonstrating the need for close clinical surveillance in these patients with a low threshold for operative management. With the potential for operative management on young patients, issues with anatomy size may pose problems. Mazur et al. demonstrated high success rates of unilateral instrumentation in congenital craniocervical instability when bilateral instrumentation was unable to be achieved [13]. Our cases further support this data. Opinions differ as to whether extensive decompression is required in these patients. Opponents state that with the lesion being chronic myelopathy, extensive decompression is not worth the risk of hematomyelia [14]. Authors who carried out C1 laminectomy or further decompression did so when there were myelopathic symptoms present, though there seems to be no real data to support this. Though Miyoshi et al. did describe a value of 12mm for SCD (sagittal canal diameter) denoting the threshold at which smaller canal diameters may require C1 laminectomy to prevent future myelopathy, the evidence was based on two cases postoperatively [4]. While research focusing on spinal canal diameter and rates of myelopathy does seem to support that smaller canal diameters when paired with the occurrence of atlantoaxial subluxation present with higher rates of myelopathy, more postoperative data is needed to support the routine performance of C1 laminectomy/other decompressive surgeries during craniocervical stabilization [4,9]. Though it is worth noting that in the cases where decompression was performed, there were no reported complications. Therefore, the potential benefit from these procedures may be weighed against minimal risks when performed by experienced surgeons.

Postoperative bracing is a topic with a minimal focus in most studies, though this is a major concern for patients clinically. Most authors in this study used invasive halo vest for three months. Complications included pain from loosening of screws and multiple pin-site infections. In our series, patients were maintained in a custom-fitted pinless halo brace with no complications and non-inferior results. In our experience, higher patient satisfaction with this mode of bracing should be taken into consideration when planning postop orthosis.

## Conclusions

Patients with SEDC are at particularly high risk of craniocervical instability. Of the patients who display craniocervical instability, a significant amount will go on to develop myelopathy if left untreated. Patients as young as three years of age have presented with myelopathy, calling attention to the need for regular surveillance and possible early operative intervention in this patient population. Our assessment of the literature has revealed no superior fusion method so long as CCJ stabilization is achieved. Decompressive surgeries during CCJ stabilization lack evidence though seem to present minimal risks. Thus, this decision should be made on a case-by-case basis by experienced surgeons.
